# The FHP01 DDX3X Helicase Inhibitor Exerts Potent Anti-Tumor Activity In Vivo in Breast Cancer Pre-Clinical Models

**DOI:** 10.3390/cancers13194830

**Published:** 2021-09-27

**Authors:** Lisa Gherardini, Giovanni Inzalaco, Francesco Imperatore, Romina D’Aurizio, Lorenzo Franci, Vincenzo Miragliotta, Adele Boccuto, Pierpaolo Calandro, Matteo Andreini, Alessia Tarditi, Mario Chiariello

**Affiliations:** 1 Istituto di Fisiologia Clinica (IFC), Consiglio Nazionale delle Ricerche (CNR), 53100 Siena, Italy; lgherardini@ifc.cnr.it (L.G.); inzalaco@student.unisi.it (G.I.); imperatore@ifc.cnr.it (F.I.); franci36@student.unisi.it (L.F.); 2 Core Research Laboratory (CRL), Istituto per lo Studio, la Prevenzione e la Rete Oncologica (ISPRO), 53100 Siena, Italy; calandro2@unisi.it; 3 Department of Medical Biotechnologies, University of Siena, 53100 Siena, Italy; boccuto2@student.unisi.it; 4 Istituto di Informatica e Telematica (IIT), Consiglio Nazionale delle Ricerche (CNR), 56124 Pisa, Italy; romina.daurizio@iit.cnr.it; 5 Department of Veterinary Sciences, University of Pisa, 56126 Pisa, Italy; vincenzo.miragliotta@unipi.it; 6 First Health Pharmaceutical B.V., 1098 XH Amsterdam, The Netherlands; matteo@firsthealthpharma.com (M.A.); alessia@firsthealthpharma.com (A.T.)

**Keywords:** triple-negative breast cancer, DDX3X, helicase, pharmacological inhibitor, WNT signaling

## Abstract

**Simple Summary:**

DDX3X has been repeatedly proven to exert pro-tumorigenic effects in breast cancer. An inhibitor of DDX3X helicase function, FHP01, exhibited very effective antiproliferative and tumor killing activity in vitro and in vivo against several breast cancer cells, yet showed no systemic toxicity but good bioavailability in vivo. FHP01 may therefore represent a novel therapeutic approach with high potential as a personalized strategy for breast cancer, confirming DDX3X as a new actionable molecular target in these tumors.

**Abstract:**

Inhibition of DDX3X expression or activity reduces proliferation in cells from various tumor tissues, in particular in breast cancer, and its expression often correlates to tumor aggressiveness. This makes DDX3X a prominent candidate for the design of drugs for novel personalized therapeutic strategies. Starting from an in silico drug discovery approach, a group of molecules has been selected by molecular docking at the RNA binding site of DDX3X. Here, the most promising among them, FHP01, was evaluated in breast cancer preclinical models. Specifically, FHP01 exhibited very effective antiproliferative and killing activity against different breast cancer cell types, among which those from triple-negative breast cancer (TNBC). Interestingly, FHP01 also inhibited WNT signaling, a key tumorigenic pathway already correlated to DDX3X functions in breast cancer model cell lines. Ultimately, FHP01 also caused a significant reduction, in vivo, in the growth of MDA MB 231-derived TNBC xenograft models. Importantly, FHP01 showed good bioavailability and no toxicity on normal peripheral blood mononuclear cells in vitro and on several mouse tissues in vivo. Overall, our data suggest that the use of FHP01 and its related compounds may represent a novel therapeutic approach with high potential against breast cancer, including the triple-negative subtype usually correlated to the most unfavorable outcomes because of the lack of available targeted therapies.

## 1. Introduction

Breast cancer is a major public health problem. It is the most frequent cancer in women and the second most common cancer overall, with over 2,000,000 newly diagnosed cases worldwide in 2020 (https://www.who.int/news-room/fact-sheets/detail/breast-cancer accessed on 20 May 2021). It is highly heterogeneous in its pathological characteristics, with some cases showing slow growth rate with excellent prognosis, while others are very aggressive tumors. Expression of estrogen and progesterone receptors (ER and PR, respectively) usually suggests a better prognosis, based on lower aggressiveness and the possibility to use endocrine therapy (tamoxifen and aromatase inhibitors) [1]. Conversely, overexpression of the HER2 protein, although allowing the use of specific drugs targeting this receptor (tyrosine kinase inhibitors or monoclonal antibodies), is usually associated with more aggressive tumors and dismal prognosis [1]. Triple-negative breast cancer (TNBC) affects approximately 10 to 20 percent of patients and is characterized by tumors that do not express ER or PR at all, and do not overexpress HER2 [1], all features making them insensitive to therapies targeting these proteins [2]. Therefore, while endocrine therapy and HER2 “inhibitors” still represent the prototype of personalized approaches for several breast tumors, new actionable molecular targets need to be continuously discovered and validated to assist clinicians in their therapeutic decisions about a considerable number of cases [3].

Cancer cells are dependent on the translational machinery due to their high demand for protein synthesis and mRNAs stabilization. DEAD-box helicases (so called because of their amino acid motif D-E-A-D, i.e., Asp-Glu-Ala-Asp) are intimately involved in these processes. They regulate RNA processing and metabolism through ATP-dependent unwinding of short double-stranded (ds) RNAs [4]. Indeed, deregulation of expression, or function, of different members of the DEAD-box protein family has been shown to be involved in biological processes controlling cancer development [5,6,7]. The *DEAD-box RNA helicase 3 X* (*DDX3X*) gene is located on the X-chromosome and is widely expressed in a broad range of organisms in which it controls pleiotropic physiological events in a variety of tissues. Specifically, DDX3X regulates different steps of RNA metabolism, including splicing, export, transcription, and translation initiation. As such, its activity has been shown to be involved in several cellular processes, from stress regulation to cell proliferation and apoptosis [8]. Based on DDX3X’s involvement in many basic mechanisms controlling cell proliferation, it is unsurprising to observe aberrant expression and function of DDX3X in human tumors. Notably, DDX3X has been repeatedly proven to exert pro-tumorigenic effects in breast cancer [8]. Specifically, knockdown of DDX3X in breast cancer cell lines decreased their proliferation and clonogenicity in vitro and reduced their ability to generate tumors and form metastases in vivo [9]. Moreover, DDX3X is often overexpressed in breast cancers, and its expression is upregulated in distant breast cancer metastases, especially in the brain, with highly metastatic DDX3X protein expression correlated to a worse survival rate [10]. Similarly, we have recently observed that women whose breast cancers have low *DDX3X* gene expression had significantly better prognosis (Log-Rank test’s *p*-value < 0.01) than the group with high expression (Figure S1), thus supporting a specific prognostic role for *DDX3X* in these tumor types. Therefore, based on all available data, DDX3X represents a promising potential target for pharmacological inhibition in breast cancers. However, none of the compounds identified that target the ATPase activity of DDX3X have progressed to clinical studies, yet. Taking advantage of in silico modeling approaches, a new class of molecules has been recently demonstrated to interact with the RNA-binding site of DDX3X [11,12], a mechanism distinct from the more conventional DDX3X inhibitors competing for the ATP binding sites. Here, we decided to study the antiproliferative activity of the FHP01 molecule [13] against human breast cancer models. Indeed, FHP01 demonstrated very strong in vitro cytotoxic effects in a panel of breast cancer cell lines, including TNBC cellular models. Interestingly, this activity correlated with its ability to suppress the WNT/β-catenin signaling axis, a pathway often correlated with DDX3X-dependent cell proliferation and transformation [14]. Based on these observations, and on the demonstration of a favorable pharmacokinetic profile and distribution in mouse models, we decided to test FHP01 activity also in a classic in vivo model for TNBC. Indeed, FHP01 demonstrated strong anti-tumor activity with no signs of specific organ toxicity and a good drug tolerance.

## 2. Materials and Methods

### 2.1. Cell Culture

Breast cancer cells were purchased from ATCC. All cells were cultured in the adequate medium at 37 °C in an atmosphere of 5% CO_2_/air. Specifically, MDA MB 231, T47D, MCF7, and 293T cells were maintained in Dulbecco’s Modified Eagle’s Medium (DMEM), 10% fetal bovine serum (FBS), 2 mM L-glutamine, 100 units per mL penicillin-streptomycin; MDA MB 468 in RPMI 1640, 10% FBS, 2 mM L-glutamine, 100 units per mL penicillin-streptomycin; SKBR3 in RPMI 1640, 10% FBS, 250 mg glucose, 1 mM sodium pyruvate, 2 mM L-glutamine, 100 units per mL penicillin-streptomycin; MCF10A in DMEM/F12, 5% Horse serum, 20 ng/mL EGF, 0.5 μg/mL Hydrocortisone, 100 ng/mL Cholera Toxin, 10 μg /mL Insulin, 2 mM L-glutamine, 100 units per mL penicillin-streptomycin. Peripheral blood mononuclear cells (PBMCs) were cultured in RPMI 1640 supplemented with 10% of heat-inactivated fetal bovine serum, 100 U/mL penicillin, 100 μg/mL streptomycin, interleukin-2 20 U/mL, HEPES 12.5 mM.

### 2.2. Helicase Assay

293T cells (3 × 10^5^) were seeded in 6-well plates and, after 24 h, transfected with 1 μg of HA-DDX3 (Addgene plasmid # 44975) [15] by using the Lipofectamine reagent (Thermo Fischer Scientific, Waltham, MA, USA). After 36 h, cells were lysed in NP-40 Lysis Buffer (20 mM HEPES pH = 7.5, 10 mM EGTA pH = 8, 40 mM β-glycerolphosphate, 1% NP40, 2.5 mM MgCl_2_, 2 mM Orthovanadate, 2 mM NaF, 1 mM DTT, and 1X protease inhibitors mixture) and 2 mg of proteins were immunoprecipitated with anti-HA antibodies (Covance, Princeton, NJ, USA) in NP40 lysis buffer. Immunoprecipitated samples were washed twice with lysis buffer and one time in helicase reaction buffer (50 mM Tris HCl pH = 8, 1 mM DTT, 5% glycerol, 10 mM MgCl_2_). Beads were then resuspended in 100 μL in helicase reaction buffer.

The helicase activity of DDX3 was tested by measuring the unwinding of an 18/36mer double stranded (ds) RNA oligonucleotide: r36mer 5′-ACCAGCUUUGUUCCUUGGGUUCUUGGGAGCAGCAGG-3′ and r18mer_FAM [6FAM]-5′-CCCAAGAACCCAAGGAAC-3′ [16]. The 36mer oligonucleotides sequence is complementary to the 18mer. An RNA oligonucleotide homologous to the 18mer (without FAM modification) was used as competitor to avoid reannealing of the probed RNA after unwinding. Annealing reactions were performed in 50 mM Tris HCl pH = 8, 10 mM MgCl_2_, 0.2 μM of r18mer, and 0.2 μM of r36mer. A sample with the final concentration of 20 nM of dsRNA plus 100 nM of the competitor were heated at 90 °C for 1 min to test efficiency of competition.

Ultimately, reactions were performed in a final volume 100 μL of helicase reaction buffer plus ATP 4 mM, 30 μL of beads used to immunoprecipitate HA-DDX3X, 20 nM of 18/36mer dsRNA, and 100 nM of competitor at 37 °C for 1 h and stopped by adding 10 μL of a stopping solution (EDTA 60 mM pH = 8, 40% glycerol, 0,6% SDS, 1% Orange G). Samples were next loaded on a 15% polyacrylamide TBE gel containing 1% SDS in 1× TBE buffer with 0.1% SDS and run at 4 °C for 1.5 h. The product of the unwinding reaction, r18mer_FAM and the dsRNA were visualized by an Image-Quant Las4000 (GE Healthcare, Chicago, IL, USA) with Cy3 detection filter and Green Epi light. Quantification of the ssRNA was performed by the ImageJ software (National Institutes of Health, Bethesda, MD, USA).

### 2.3. Western Blots

Cells (1 × 10^5^) were seeded in 6-well plates. After 24 h, cells were treated for indicated times with DMSO (vehicle) or FHP01 or XAV939 (Merck). After treatments, cells were washed with cold PBS and total protein extracts obtained by adding 80 µL of “RIPA Lysis buffer” (50 mM Tris-HCl pH 8.0, 150 mM NaCl, 0.5% Sodium deoxycholate, 0.1% SDS, 1% NP-40, 2 mM Orthovanadate, 2mM Sodium fluoride, 1mM Dithiothreitol and 1× protease inhibitors). After mechanicals detachment with cell scrapers, total lysates were collected in tubes, vortexed, and incubated for 15 min on ice. Tumors from mice were collected and disrupted by bead homogenization in Lysing Matrix D tubes using a FastPrep-24 5G homogenizer (MP Biomedicals, Solon, OH, USA) filled with “RIPA Lysis buffer” w:v. Next, samples were centrifuged for 10 min at 16,000× *g*, and supernatants were collected, representing total cell lysates.

For Western blot analysis, 20 μg of proteins derived from total lysates was loaded on 8% polyacrylamide gels with 1× of Laemmli buffer and resolved by SDS-PAGE, transferred to Immobilon-P PVDF membrane (Millipore, IPVH00010), probed with anti-DDX3 (Cell Signaling, 2635), anti-β-catenin (c19220, BD Biosciences, Franklin Lakes, NJ, USA), anti-phospho β-catenin (9561, Cell Signaling, Danvers, MA, USA), anti-ERK2 (sc-154, Santa Cruz, Dallas, TX, USA), and revealed by enhanced chemiluminescence detection (ECL Plus; GE Healthcare, RPN2132). Densitometric analysis of western blots was performed with NIH Image J (National Institutes of Health).

### 2.4. Luciferase Assays

MDA MB 231 cells were seeded at 3 × 10^4^ cells/well density in 12-well plates, in triplicate, and transfected with 250 ng of the M50 Super 8× TOPFlash reporter vector (Addgene Plasmid #12456) [17] and 1 μg of expression vectors inducing the WNT pathway (Wnt1-V5, Addgene plasmid #43807, and Wnt3A-V5, Addgene plasmid #43810) [18]. Eight hours after transfection, cells were treated with FHP01, XAV939 (Merck, Kenilworth, NJ, USA) or DMSO for indicated times prior to lysis in Passive Lysis Buffer (Promega, Madison, WI, USA). Luciferase activity in cellular lysates was assessed on a Glomax 20/20 luminometer (Promega) using the Luciferase Assay System (Promega). Results were normalized for GFP expression, upon transfection with GFP coding vector (1 μg). Representative results from 3 independent experiments are shown (*n* = 3). Error bars represent the Standard Error of the Mean. All samples were read at least in triplicate.

### 2.5. Cell Viability Assays in Cancer Cells

Cells were seeded at 3–5 × 10^4^ cells/well density (depending on cell lines) in 12-well plates, in triplicate, and maintained at 37 °C in 5% CO_2_. After 24 h, cells were treated with different concentrations of indicated drugs in a range from 0 to 100 μM (0, 0.1, 1, 10, 50, 100 μM). After 72 h incubation, cells were washed in PBS and harvested. Cell number of each sample was determined with Z2 Coulter Counter (Beckman Coulter, Brea, CA, USA), in triplicate. Data about cell viability were plotted in GraphPad Prism 8.0 software (GraphPad Software, San Diego, CA, USA) to draw dose-response curve and to calculate IC_50_.

### 2.6. Cell Viability Assays in Peripheral Blood Mononuclear Cells (PBMCs)

Two days before the assay, phytohemagglutinin-A (PHA) 2 µg/mL was added to the PBMC culture. Cells were seeded at 13 × 10^4^ cells/well in triplicate and maintained at 37 °C in 5% CO_2_. After 24 h, cells were treated with 10 μM and 50 μM of indicated drugs. After 72 h incubation, cells were washed in PBS and harvested. Cell number of each sample was determined with Z2 Coulter Counter (Beckman Coulter), in triplicate. Data about cell viability were plotted in GraphPad Prism 8.0 software 100% viability corresponding to vehicle (DMSO) treated PBMCs.

### 2.7. Pharmacokinetic Studies in Animal

These studies were performed at the GVKBioscience Pvt. LTD Contract Research and Development Organization (CRDO). Animals were housed in cages with clean bedding. Adult Swiss Albino male mice (weight = 20–35 g) were maintained and monitored for good health in accordance with GVKBioscience Pvt. LTD Test Facility SOPs and at the discretion of the laboratory animal veterinarian. Certified rodent diet was provided, and water was available ad libitum. A periodic analysis of the water was performed, and the results have been archived at the Test Facility. Environmental controls for the animal room were set to maintain a temperature of 22 to 25 °C, humidity of 40–70% relative humidity, and a 12-h light/12-h dark cycle. Animals have been dosed i.v. with 1 mg/kg of FHP01 i.p. with 10 mg/kg of compound or p.o. through gastric gavage needle with 50 mg/kg of compound. Next, mice were anesthetized using gaseous anesthesia and blood samples were collected at each time point, from 3 mice of respective group. After collection of blood samples at each time point, the blood samples were stored on ice, prior to centrifugation. Blood samples were centrifuged at 1540 rcf (5000 rpm), 4 °C for 10 min to separate plasma. Plasma was transferred to pre-labeled micro-centrifuge tubes and promptly frozen at −80 °C until analysis. Samples have been identified by test item, group, animal number, and collection time point. A fit-for-purpose bioanalytical method was developed for analyzing the plasma samples. One set of nine standards have been run before the sample batch and used for plotting the calibration curve. Quality control (QC) samples have been prepared at a minimum of three concentrations, i.e., LQC (not more than 5 times to that of lowest standard concentration), HQC (not less than 75% of the highest standard concentration), and MQC (between the low and high concentration). Minimum of 6 QC samples (three concentrations in duplicate). One Set of QC (LQC, MQC, and HQC) samples have been analyzed before and after the sample batch.

For the analyses of drug distribution to brain and liver, whole body perfusion was performed using chilled saline in the defined group of animals followed by decapitation for tissue collection. The chest of the mice was exposed, the abdominal aorta was clamped, and both jugular veins were cut. Intra-cardial perfusion was performed through an insertion in the left ventricle. Mice brains and livers have been collected and immediately washed, dried, weighed and freeze at −80 °C until homogenization. The protocol has been reviewed and approved by the Institutional Animal Ethics Committee (IAEC).

### 2.8. Cancer Model Animal Study

Animal experiments were performed in accordance with current Italian legislation and the animal protocol has been reviewed and approved by Italian Ministero della Salute (authorization n. 886/2020-PR). The study was designed to verify the effect of newly synthetized molecule with DDX3X inhibitor activity administered via i.p at a concentration of 45 mg/kg on the tumor growth of xenograft MDA MB 231 human breast cancer cells. In the study, n refers to the number of tumors expressed on both flanks of the animals. The sample size was calculated by g-power calculator placing a predictable large (2-fold increase) effect size, alpha as 0.05, and a confidence interval of 95% and we set our experimental unit number of 10 (corresponding to 5 animals). Total number of animals used in the study was 10. Six-week-old athymic nude mice were purchased from Charles River Laboratories. Five animals in each cage acclimatized in our animal facilities for 5 days in individually ventilated cages. Animals were fed with the lab diet ad libitum and water was accessible at all times and kept under standard specific pathogen-free conditions of 12 h light/dark cycle. Observational analysis was performed at the beginning of experimental procedure to ensure animal wellbeing (Table S1).

To establish xenograft tumors, MDA MB 231 cells were cultured in media as described above. On the day of experiment, cells (2.8 × 10^6^) were suspended in 150 μL PBS 1:1 Matrigel and injected subcutaneously on both size of the flank region of nude mice. In order to determine tumor volume by manual caliper, the greatest longitudinal diameter (length) and the greatest transverse diameter (width) were determined. Tumor volumes based on caliper measurements were calculated by the modified ellipsoidal formula Tumor volume = 1/2(length × width^2^).

Group allocation and treatments: once tumors reached a mean size of 24.82 ± 2.56 mm^3^, animals were allocated in two groups by minimization strategy according to tumor size: the treatment group received 100 μL of a 45 mg/kg FHP01 suspended in a vehicle consisting of 10% DMSO, 5% Tween 80, 85% H_2_O via i.p. injection three times per week for 4 weeks; the control group included animals treated with 100 μL of vehicle (10% DMSO, 5% Tween 80, 85% H_2_O). One control animal reached HEP and was sacrificed and was not account for in the analysis. The FHP01 treatment group showed an outlier that was not accounted for. At the end of the experiment, animals were euthanized according to FELASA protocol and internal organs of interest were collected for postmortem histology.

For the final statistics, the number of tumors analyzed was 8 in the control group (one animal died for unknown reasons as certified by the AWB designed veterinary) and 9 for the treated group (the volume of one tumor was considered outlier). Results were reported as tumor volume increase, normalizing the value of each measurement to the baseline value of each sample and expressed as mean ± SEM. Two-way RM ANOVA was used to describe the significant effect of treatment versus time, considered significant when *p* < 0.05.

### 2.9. Histology Sampling

Animals were euthanized and internal organ samples collected. Spleen, kidney, lung, and brain from each accounted animal were formalin fixed and paraffin embedded. H&E staining of 4-µm-thick sections was performed to unveil sign of toxicity and loss of morphology.

### 2.10. Survival Analysis

Gene expression and clinical data were retrieved from GEO database (accession number [GSE31519], https://www.ncbi.nlm.nih.gov/geo/query/acc.cgi?acc=GSE31519 accessed on 21 August 2020). We analyzed DDX3X probe specific expression vs. Event-Free Survival. After splitting patients according to DDX3X median expression level of 4 probes, we performed survival analysis using R software (Version R-4.0.1, R Foundation for Statistical Computing, Vienna, Austria) [19]. Specifically, Kaplan-Meier survival curves were built, visualized, and compared (through Log-rank test) by using “survival” and “surviminer” CRAN packages.

### 2.11. Statistical Analysis

For each analysis, data are represented as the mean ± SEM. For comparison, statistical significance was tested using t-test for selected time point. Two-way RM ANOVA was applied to verify the effect of the two treatments in vivo. All *p*-values were based on two-sided statistical analyses, and *p* < 0.05 was considered statistically significant.

## 3. Results

### 3.1. FHP01 Treatment Exerts Strong Cytotoxic Activity on TNBC Cells

**Figure 1:**
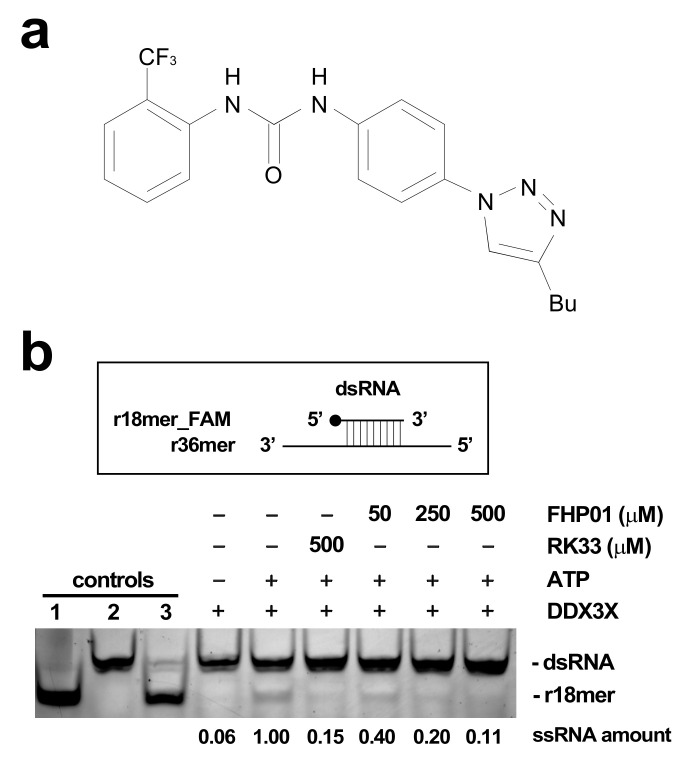
(**a**) FHP01 compound. (**b**) FHP01 helicase assay. Upper panel: the assay scores the ability of immunoprecipitated HA-DDX3X of unwinding a previously annealed dsRNA constituted by a fluorescein amidites (FAM)-modified r18mer and a complementary r36mer. Lower panel: the products of unwinding reaction were run on a polyacrylamide gel to distinguish them from the dsRNA. Unwinding reactions were run in presence of the vehicle (+ATP) or indicated amounts of the RK33 and FHP01 DDX3X inhibitors. A complete reaction without ATP (−ATP) was also run to ascertain background level of activity in this assay. As additional controls, the FAM-conjugated r18mer (1), the dsRNA (2), and a dsRNA annealed in excess of a r18mer unconjugated to FAM (competitor) (3) were also loaded. The r18-mer_FAM product of unwinding reaction was quantified by the ImageJ software. The result of a representative helicase assay experiment is shown (*n* = 3).

FHP01 (Figure 1a) is an inhibitor of DDX3X helicase activity (in vitro enzymatic activity, IC_50_ = 0.3 μM) [13] and belongs to a class of molecules discovered and optimized through an in silico drug design approach, by means of 3D-pharmacophore screening and molecular docking calculations to identify compounds targeting the RNA binding site of DDX3X [11,12,13]. Indeed, we validated the binding mode of FHP01 by performing a homology modeling of DDX3X and a molecular dynamic simulation (Figure S2). Furthermore, as DDX3X is emerging as a very complex protein whose activity may be influenced by several prost-translational modifications [20], which cannot be properly modeled by bacterially overexpressed proteins, we decided to confirm its ability of inhibiting DDX3X helicase activity using the human protein immunoprecipitated from 293T cells transfected with HA-tagged DDX3X in a qualitative in vitro helicase assay, demonstrating an activity comparable to RK33 (Figure 1b, The original Western blot images have been shown in Figure S9), a lead compound inhibitor for DDX3X [21].

**Figure 2:**
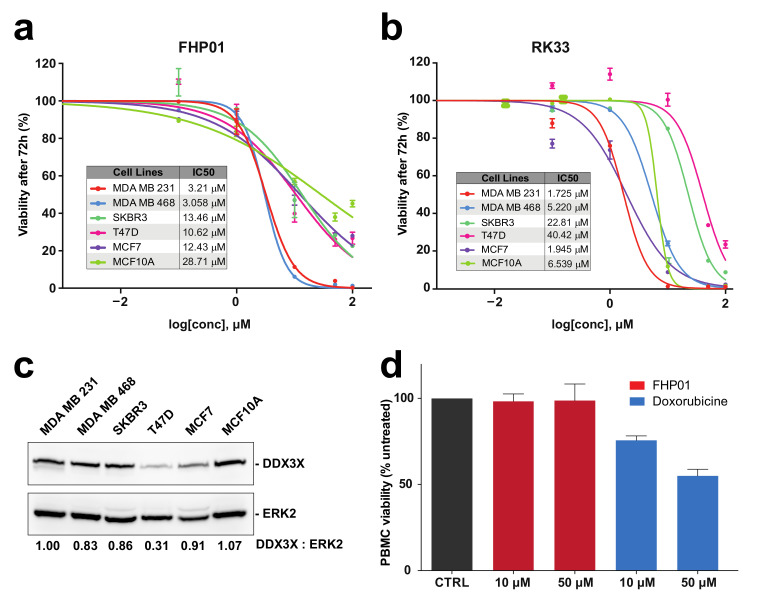
Cytotoxicity evaluation in different human breast-derived cell lines. (**a**) IC_50_ evaluation, after 72 h treatment of MDA MB 231, MDA MB 468, SKBR3, T47D, MCF7 breast cancer cells and control MCF-10A immortalized cells, with the FHP01 compound, at different concentrations (0, 0.1, 1, 10, 50, 100 μM), obtained by cell counting through a Z2 coulter counter. The results shown are averages of triplicate samples from a typical experiment; (**b**) Same as in panel (**a**) but using the RK33 molecule; (**c**) DDX3X expression in different breast cancer cell lines, evaluated by western blot analysis. Anti-ERK2 immunoblot was used to normalize total lysates protein levels. Representative images from 3 independent experiments are shown (*n* = 3). Intensitometric analysis of DDX3X, normalized by ERK2 protein levels, was performed using NIH ImageJ; (**d**) Cell viability of PBMCs was measured after 72 h treatment with indicated concentrations of FHP01 and Doxorubicin (used as a positive control).

Based on the reported key role of DDX3X in breast cancer [9,22], we decided to test FHP01 efficacy in vitro by measuring its IC_50_ values in different cell lines representing models of the principal molecular subtypes of breast cancer: MCF7 and T47D (ER+/PR+ subtype), SKBR3 (HER2+ subtype), MDA MB 468 and MDA MB 231 (TNBC subtype), and the immortalized MCF10A cells as non-tumorigenic mammary epithelial control cells (Figure S3) [2]. Specifically, in this cytotoxicity assay, after 72 h of continuous incubation with cells, FHP01 efficacy was higher in TNBC cells (IC_50_ = 3.058 and 3.21 μM in MDA MB 468 and MDA MB 231, respectively) intermediate in ER+/PR+ (IC_50_ = 12.43 and 10.62 μM in MCF7 and T47D cells, respectively) and HER2+ (IC_50_ = 13.46 μM in SKBR3) cells, but lower in control MCF10A cells (IC_50_ = 28.71 μM) (Figure 2a). Noteworthy, efficacy of FHP01 in breast cancer cellular in vitro models was comparable to RK33 (Figure 2b) and was not strictly correlated to DDX3X expression (Figure 2c), as already shown for RK33 itself [23]. Importantly, our data strictly phenocopied the already reported effect of DDX3X knockdown in breast tumor cells [9], confirming very high potential for this gene as a specific target for breast cancer therapy. As an additional control for the ability of the FHP01 compound to specifically affect cancer cell survival, no effect of the drug was observed on normal peripheral blood mononuclear cells (PBMC) up to a concentration of 50 μM (Figure 2d). Ultimately, we verified the effects of the drug on the two best performing models, MDA MB 231 and MDA MB 468 TNBC cells, also after 24 and 48 h incubations, demonstrating progressively decreasing IC_50_ values in both cases at the different time-points (Figure S4).

### 3.2. FHP01 Inhibits WNT Signaling in TNBC Cells

**Figure 3:**
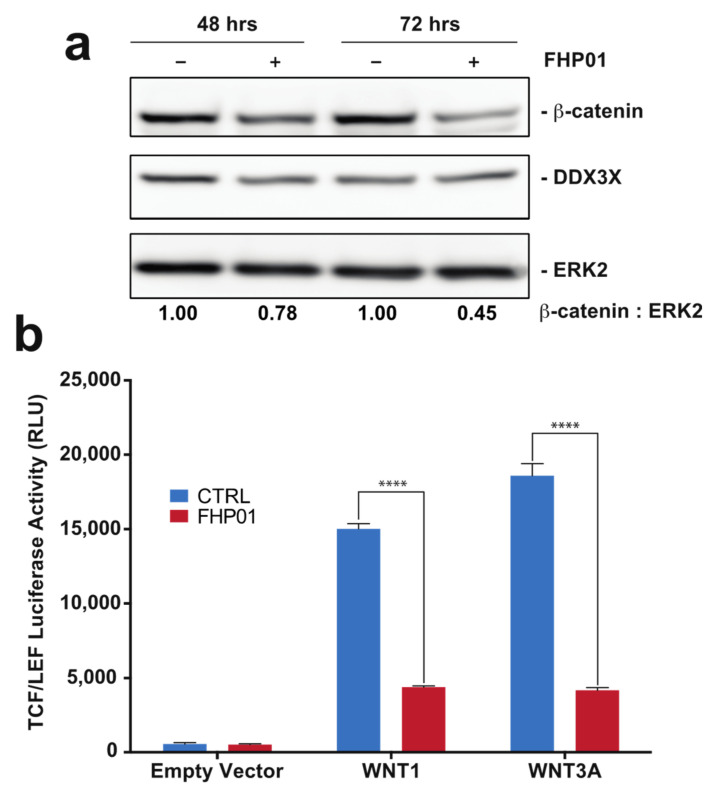
FHP01 inhibits WNT signaling. (**a**) MDA MB 231 cells were incubated with vehicle or the FHP01 DDX3X inhibitor (6 μM) for indicated times and then immunoblotted to score β-catenin and DDX3X protein levels. Anti-ERK2 immunoblot was also used to normalize total lysates protein levels. Representative images from 3 independent experiments are shown (*n* = 3). Intensitometric analysis of β-catenin, normalized by ERK2 protein levels, was performed using NIH ImageJ; (**b**) activation of TCF/LEF luciferase reporter vector after transfection with plasmids coding for the soluble ligands WNT1 and WNT3A, and 6 μM FHP01 treatment (48 h), as indicated. RLU, relative light units. **** *p* < 0.0001.

Since the first evidence of a role of the DDX3X protein as a regulator of the WNT-β-catenin signaling in mammalian cells and during Xenopus and *Caenorhabditis elegans* development [24], several reports have demonstrated an involvement of the DDX3X-WNT-β-catenin axis in different human tumors [21,25,26]. Importantly, DDX3X’s ability to control β-catenin expression appears to require its RNA helicase activity [25]. Based on these data, we decided to use the activation of the WNT-β-catenin pathway as an alternative, although indirect, readout to demonstrate that FHP01 acts on DDX3X, being able to down-regulate the activation of the WNT pathway downstream of this RNA helicase. To this aim, we first performed Western blot analysis of MDA MB 231 cells treated from 48 to 72 h with FHP01 and observed a time-dependent reduction of β-catenin protein levels (Figure 3a), an event already described in relation to the downregulation of WNT signaling upon DDX3X knockdown {Chen et al., 2015, #17808}. As an additional control for this experiment, an established inhibitor of WNT signaling in breast cancer cells, XAV939 [27], was also used in the same cellular model, demonstrating a significant increase of β-catenin phosphorylation (Figure S5a), as already previously demonstrated [28]. Next, to confirm these data also in a WNT pathway functional assay, we took advantage of the availability of a T cell factor/lymphoid enhancer factor (TCF/LEF) luciferase reporter assay, TCF/LEF transcription factors representing the major end-point mediators of WNT signaling throughout metazoans. Specifically, we observed strong inhibition of WNT1- and WNT3A-dependent TCF/LEF transcriptional activity [18] when treating cells with FHP01 (Figure 3b), therefore confirming the already reported efficacy of DDX3X inhibition in down-regulating the activation of the WNT pathway. Further, in this case, the XAV939 WNT inhibitor was used as a control, demonstrating efficient attenuation of TCF/LEF-dependent transcription in the same experimental settings (Figure S5b), overall showing that the phenotype of the two drugs reproduce each other, ultimately supporting our experimental hypothesis.

### 3.3. Efficient In Vivo Anti-Tumor Activity of FHP01 in a Preclinical Mouse Model of TNBC

As our in vitro data suggest that the therapy of breast cancer, and more specifically of TNBCs, the most aggressive subtype of breast cancer, may benefit from the antitumor activity of DDX3X inhibitors, we next set up to study the efficacy of FHP01 treatment in vivo, in a TNBC mouse model. To this aim, we first tested which route of administration, i.e., intravenous (i.v.), intraperitoneal (i.p.), and per os (p.o.), could perform best in mice after acute administration, to ensure bioavailability of the drug. Indeed, low/absent efficacy in vivo of other DDX3X inhibitors targeting the ATP-binding site has been previously ascribed to the lack of efficient drug delivery, rapid clearance, and drug degradation [9]. Importantly, we found that all tested individual routes of administration were suitable to reach sufficient drug plasma concentrations to exert antiproliferative effects on tumor cells in vivo (Figure S6a). Specifically, while a fast kinetic was observed after i.v. injection, with a rapidly decreasing plasma concentration (Figure S6a, red line), both i.p. (Figure S6a, blue line) and p.o. (Figure S6a, green line) administration showed a high plasma concentration, resembling in vitro IC_50_, which remained relatively stable for several hours. Moreover, while it was quite expected to find high level of FHP01 distribution into the liver, it was interesting to note high levels of distribution also into the brain at 8 h after i.p. injection (Figure S6b), thus suggesting elevated bioavailability of the drug even in difficult to reach tissues such as those beyond the blood brain barrier and supporting the use of i.p. administration for in vivo experiments. Ultimately, FHP01 tissue concentration rapidly decreased below the level of quantitation at 24 h after administration, thus ruling out the risks of undesired accumulation into the organs (Figure S6b).

**Figure 4:**
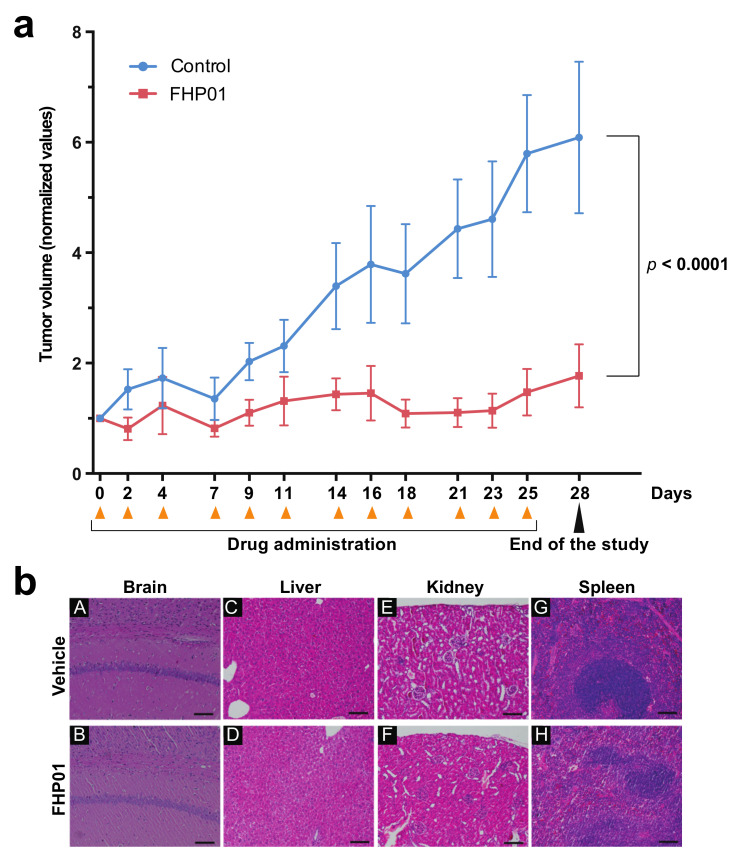
Growth of MDA MB 231 tumor xenografts in nude mice is suppressed in response to FHP01 treatment. (**a**) Athymic nude mice were injected with MDA MB 231 on both flanks. Once tumor volume reached approximately a mean size of 25.42 ± 2.02 mm^3^, animals were treated with 45 mg/kg FHP01 suspension i.p. three times per week for 4 weeks. Average tumor volume of control (blue, *n* = 8) significantly diverges from the volumes recorded in FHP01 treated (red, *n* = 9) nude mice. The data of the volumes are normalized to the baseline value of each sample (normalized values) and expressed as mean ± SEM. Comparisons were subjected to two-way RM ANOVA. (**b**) Representative images of organ histological investigation after 4 weeks, three time per week, of i.p. treatment with FHP01 (45 mg/kg). Qualitative analysis did not detect any evident signs of toxicity in brain (A,B), liver (C,D), kidney (E,F), and spleen (G,H) of controls (A,C,E,G) and treated (B,D,F,H) animals (scale bars are equal to 100 µm).

To evaluate FHP01-induced in vivo antitumor effects, we decided to use a well-established preclinical animal model for human TNBCs, using athymic nude mice bearing subcutaneous MDA MB 231 human cell-derived tumor xenografts. Two weeks after the grafting of the tumors, they were measured (mean tumor volume 24.82 ± 2.56 mm^3^) and animals were randomized and assigned by minimization to two different groups and treated with FHP01 (45 mg/kg, i.p. injection, 3 times a week) or with the same volume of vehicle as a control. Results indicated that FHP01 treatment exerted a significant in vivo anti-tumor effect when compared to the control group (Figure 4a). Specifically, at Day 0 of the treatment, mean tumor volume of the FHP01 group (25.49± 4.58 mm^3^) was similar to that in the vehicle group (24.01 ± 3.64 mm^3^). Conversely, starting from Day 18, tumor volumes were significantly different between the two treatment groups (24.18 ± 4.44 mm^3^ in the FHP01 group vs. 73.40 ± 9.72 mm^3^ in the control group). This difference continued to increase until the end of the experiment at Day 28 (37.23 ± 11.45 mm^3^ in the FHP01 group vs. 129.85 ± 21.39 mm^3^ in the control group) (Figure 4a). A significant interaction between treatment and time was asserted with two-way RM ANOVA analysis (*p* < 0.0001). No loss of animal body weight was detected during all the treatment period (Figure S7) nor other physical or behavioral alterations were observed, indicating a general state of good health of all injected animals with an overall excellent biocompatibility of the drug and a good tolerance of nude mice to the dose of 45 mg/kg. The effect size calculated at the end of the procedure was about 4-fold decrease in tumor growth (control/treated) with a confidence interval of 95%. These results are considered extremely relevant for future translational implementation of the treatment with FHP01 in human triple-negative breast cancer.

At the end of the treatments, animals were sacrificed and histological analysis of liver, spleen, brain, and kidney from mice belonging to both groups of the efficacy study was performed, to evaluate whether treatment-related toxicity of the DDX3X inhibitor might be present. Post-mortem investigation did not show any sign of toxicity in liver, brain, spleen, and kidney, after 4 weeks of i.p. treatment with FHP01 (Figure 4b), despite the good biodistribution to different organs and the significant antitumor effects obtained on the human TNBC-derived tumors. Furthermore, three independent tumors for each condition were analyzed for the expression of DDX3X and β-catenin, demonstrating a strong reduction of DDX3X protein levels in tumors upon prolonged administration of FHP01 and a consistent reduction of β-catenin in the same samples (Figure S8), overall supporting a specific action of the drug on DDX3X also in vivo.

## 4. Discussion

Altogether, our results clearly indicate that the FHP01 compound exerts powerful in vivo anticancer activity as a single agent against a TNBC tumor model. In turn, this supports the possibility of using DDX3X helicase inhibitors against breast cancer. Nonetheless, while DDX3X has a clear pro-tumorigenic role in breast cancer, there are examples of context-dependent pro- or anti-proliferative effects for DDX3X in different or even in the same cancer types [7,8]. This aspect needs, therefore, to be considered in the hypothesis of using DDX3X inhibitors for cancer therapy in humans. In this context, DDX3X mutations have also been identified in some cancer types, i.e., medulloblastomas, head and neck squamous cell carcinomas (HNSCC), and hematological malignancies [6]. Specifically, in medulloblastomas and natural killer/T cell lymphoma (NKTCL), DDX3X represents one of the most frequently mutated genes [29,30]. Unfortunately, the effects of these mutations on DDX3X functions are still debated [29,30,31], making it difficult to define whether inhibition rather than stimulation of the DDX3X activity would be necessary to target these specific tumors.

Based on their potential anticancer and antiviral effects, multiple DDX3X inhibitors have been identified and studied over the last 10–15 years [8,32]. As an ATP-dependent RNA helicase, DDX3X offers two main mechanisms that can be exploited for inhibition: direct inhibition at the ATPase domain or, alternatively, occupation of the RNA-binding site. Drugs targeting the ATP-binding site block both the ATPase and helicase activity of DDX3X, therefore representing an efficient method to impair DDX3X functions. However, ATP-binding sites are present in several classes of enzymes with a consequent intrinsic potential for non-specific binding to these proteins, and increased probability of toxic effects. Conversely, drugs targeting RNA binding to the helicase domain will likely represent a more selective class of inhibitors with a narrower pharmacological profile: nonetheless, most current DDX3X inhibitors mostly target its ATP binding site [8,32]. Unfortunately, while these ATP binding site inhibitors demonstrate efficient cytotoxicity in vitro, their single agent efficacy in vivo is poor. The only effects observed are, in fact, often limited to a radio-sensitizing effect, as shown previously in breast cancer preclinical models, possibly because of selectivity or bioavailability issues [8,32,33]. Conversely, all of them showed low toxicity in in vivo models, suggesting that inhibition of DDX3X activity per se is not toxic and that drugs with improved physicochemical and pharmacodynamic characteristics might represent a promising approach for breast cancer treatment. In fact, more selective DDX3X inhibitors that solely target its helicase activity have been recently identified and shown to exert strong antiviral efficacy and low toxicity [11]. Consequently, we hypothesized that these types of drugs could represent good candidates to inhibit breast cancer cells’ proliferation with a novel targeted approach. Here, we demonstrated for the first time that, indeed, FHP01 showed a significant antiproliferative effect against several human breast cancer cells and this effect was particularly evident for TNBC cells. As TNBC is the subtype with the most unfavorable outcomes, the need for new targets is paramount [2]. Importantly, the effect of in vivo FHP01 treatment, as a single agent, in TNBC mouse models revealed very high efficacy and low toxicity, overall demonstrating a strong potential for this class of molecules as a more effective therapeutic approach for this very aggressive type of cancer.

## 5. Conclusions

Nowadays, there is a huge interest in identifying new actionable molecular targets for personalized medicine. Among them, DDX3X has already demonstrated good potential for targeted pharmacological approaches in breast cancer, based on its pro-tumorigenic role in these tumors. Here, we show that FHP01 exerts very effective antiproliferative and killing activity against different breast cancer cell types and strongly inhibits tumor growth in xenograft models, supporting the hypothesis that targeted inhibition of DDX3X helicase activity is potentially exploitable in breast cancer patients and may positively affect the prognosis of this disease.

## Data Availability

Gene expression and clinical data used in this study were obtained from GEO database (www.ncbi.nlm.nih.gov/geo/query/acc.cgi?acc=GSE31519 accessed on 21 August 2020).

## Supplementary Materials

The following are available online at https://www.mdpi.com/article/10.3390/cancers13194830/s1, Figure S1: Kaplan-Meier curves for Event Free Survival of TNBC patients from GSE31519 dataset, Figure S2: Best score docking poses of FHP01 in DDX3X RNA binding site, Figure S3: Molecular characteristics of different human breast cancer-derived cell lines, Figure S4: Cytotoxicity evaluation in TNBC cells after 24 and 48 h incubation with FHP01, Figure S5: XAV939 inhibits WNT signaling, Figure S6: Pharmacokinetic profiling and concentrations in specific tissues of FHP01, Figure S7: Assessment of animal wellbeing, Figure S8: FHP01 reduces DDX3X expression and WNT signaling in tumors from treated mice, Figure S9: Original Western Blot images, Figure S10: Homology Modeling, Figure S11: Sequence alignment of the DDX3X sequence (Target) with the template sequence (2db3.1.B), Figure S12: RMSD variation of carbon alpha trace during the Molecular dynamics simulation of DDX3X, Figure S13: List of residues composing the RNA binding site DDX3X, Figure S14: Sorted RMSD distance matrix between conformations of DDX3X obtained from the molecular dynamic simulation, Table S1. ARRIVE-study in vivo guideline grid. Supplementary Materials and methods: Homology Modeling, Molecular Dynamics Simulations, Ensemble Molecular Docking, Synthesis of FHP01.

## Abbreviations

DDX	DEAD-box RNA Helicases
TNBC	triple-negative breast cancer
IC_50_	half-maximal inhibitory concentration
ER	estrogen receptor
PR	progesterone receptor
HER2	human epidermal growth factor receptor 2
TCF/LEF	T cell factor/lymphoid enhancer factor
i.v.	intra-venous
i.p.	intra-peritoneal
p.o.	per os.
