# Supplementary material for: The FHP01 DDX3X Helicase Inhibitor Exerts Potent Anti-Tumor Activity In Vivo in Breast Cancer Pre-Clinical Models
Source: Cancers (Basel). 2021 Sep 27;13(19):4830. doi: 10.3390/cancers13194830 (PMC8507746; doi:10.3390/cancers13194830)
Supplement: Supplementary file 1 [file cancers-13-04830-s001.zip › cancers-1404031-supplementary.pdf]

Supplementary Materials: The FHP01 DDX3X Helicase Inhibitor Exerts Potent Anti-Tumor Activity In Vivo in Breast Cancer Pre-Clinical Models

Lisa Gherardini, Giovanni Inzalaco, Francesco Imperatore, Romina D'Aurizio, Lorenzo Franci, Vincenzo Miragliotta, Adele Boccuto, Pierpaolo Calandro, Matteo Andreini, Alessia Tarditi and Mario Chiariello

1. Supplementary Figures

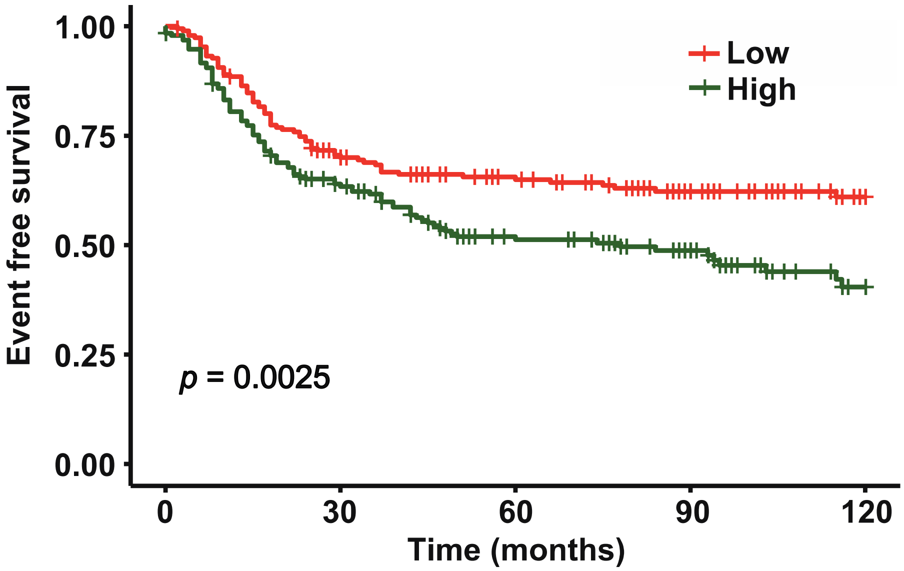

Figure S1. Kaplan-Meier curves for Event Free Survival of TNBC patients from GSE31519 dataset. To investigate the impact of DDX3X gene expression on the clinical outcome of TNBC patients, we exploited a cohort of 579 cases and event free survival (EFS) follow up data, corresponding to relapse-free survival endpoint. Samples are divided in two groups according to the DDX3X median expression level ("Low" stands for < median value; "High" stands for > median value). Log-rank test's *p*-value is shown.

a

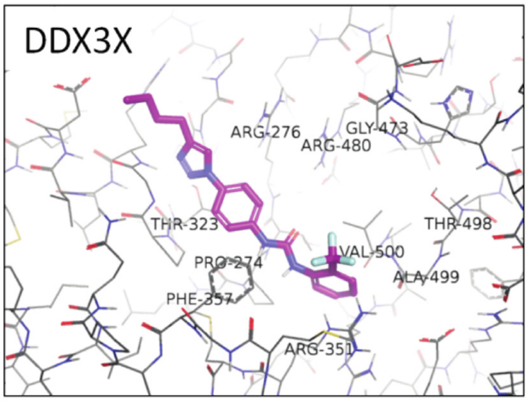

b

Interaction type - index	Residue interacting part	Binding site residues index
HBD-1	Sidechain	Arg 276
HBD-2	Sidechain	Arg 480
HBD-3	Sidechain	Thr 498
VdW/HBA-4	Backbone	Pro 274
VdW-5	Sidechain	Phe 357
VdW-6	Sidechain	Val 500
VdW-7	Sidechain	Gly 473
HBA/HBD-8	Sidechain	Thr 323
HBD-9	Sidechain	Arg 351
HBA/D/VdW-10	Sidechain	Ala 499

Figure S2. Best score docking poses of FHP01 in DDX3X RNA binding site. The DEAD box helicases consist of two main RecA-like domains that can change their reciprocal position upon the binding of an ATP/ADP co-factor and/or double strand RNA. The reciprocal positions of the two domains are usually indicated as "open" and "closed" conformations. The closed conformation is identified with the binding of double stranded RNA and ATP/ADP, while the open conformation with the binding of only ATP/ADP. Most of the available X-Ray structures of the human DEAD box helicases were obtained in the open conformation. To independently validate the binding mode of FHP01 against the previous published data for this compound series [1] we performed a homology modeling of DDX3X and a molecular dynamic simulation with the objective to collect a set of protein conformations suitable for an ensemble docking procedure. **(a)** The application of our molecular docking protocol on multi conformation DDX3X ensemble docking model allow to guess a concrete hypothesis about the binding mode of FHP01 in the RNA binding site. **(b)** The best score binding pose obtained (RFScore of 7.33) showed a set of key interaction with the RNA binding site [2] that are in agreement with the binding mode proposed by Brai et al. for the compound series which FHP01 belongs [1].

Cell line	ERα	PR	HER2	Other characteristics
MCF-10A	–	–	–	Immortalized and non-tumorigenic cell line
MCF-7	+	+	–	Model for endocrine responsive breast cancer
T47D	+	+	–	Model for endocrine responsive breast cancer
SK-BR-3	–	–	+	Model for HER2 positive breast cancer
MDA-MB-468	–	–	–	Model for triple negative breast cancer
MDA-MB-321	–	–	–	Model for triple negative breast cancer

Figure S3. Molecular characteristics of different human breast cancer-derived cell lines. ER, estrogen receptor; HER2, human epidermal growth factor receptor 2; PR, progesterone receptor.

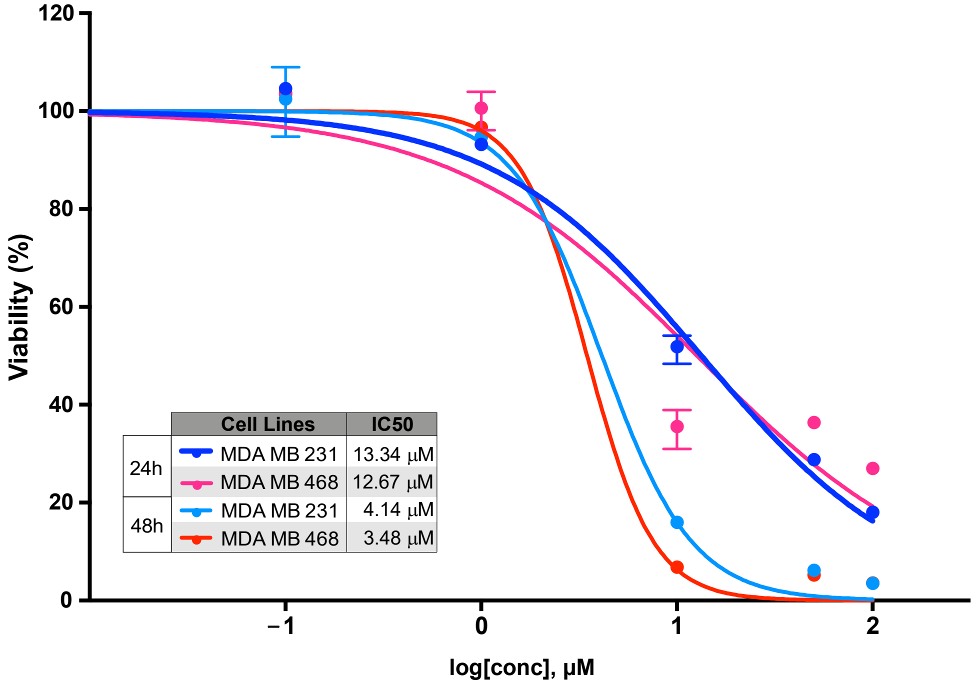

Figure S4. Cytotoxicity evaluation in TNBC cells after 24 and 48 hrs incubation with FHP01. IC_{50} evaluation, after 24 and 48 hrs treatment of MDA MB 231 and MDA MB 468 breast cancer cells with the FHP01 compound, at different concentrations (0, 0.1, 1, 10, 50, 100 μM), obtained by cell counting through a Z2 coulter counter. The results shown are averages of triplicate samples from a typical experiment.

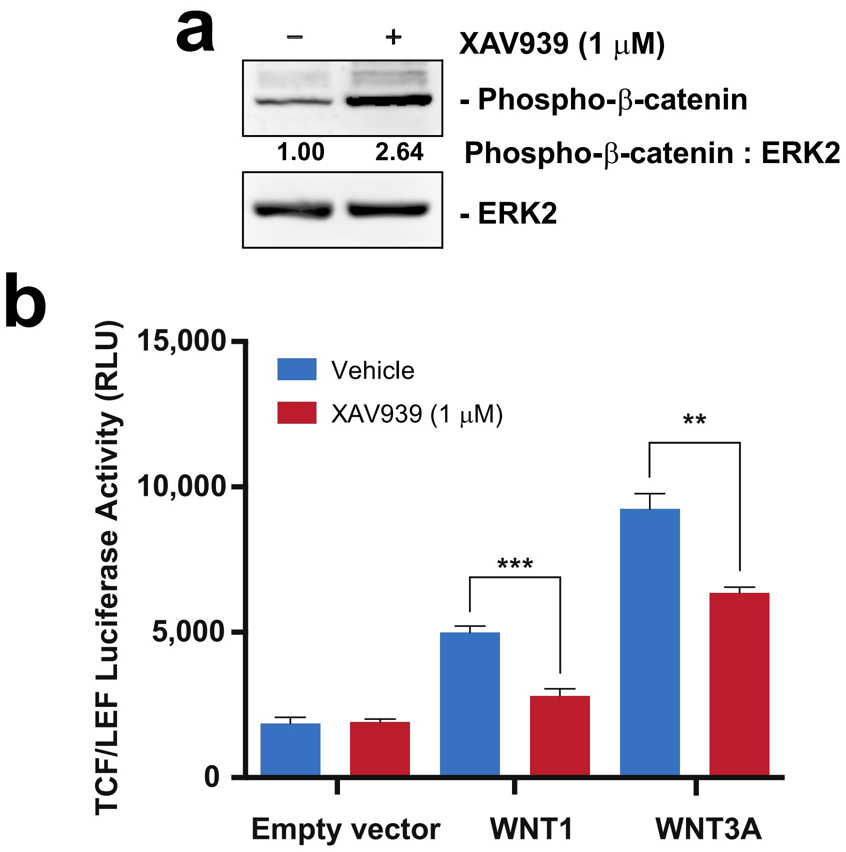

Figure S5. XAV939 inhibits WNT signaling. **(a)** MDA MB 231 cells were incubated with vehicle or the XAV939 WNT inhibitor (1 μM, 16 hrs) and then immuno-blotted to score phosphor-β-catenin protein levels. Anti-ERK2 immunoblot was also used for normalization purposes. Intensitometric analysis of phosphor-β-catenin, normalized by ERK2 protein levels, was performed using NIH ImageJ; **(b)** Activation of TCF/LEF luciferase reporter vector after transfection of MDA MB 231 with plasmids coding for the soluble ligands WNT1 and WNT3A, and XAV939 treatment (1 μM, 16 hrs). RLU, relative light units. ***p* < 0.01; ****p* < 0.001.

a

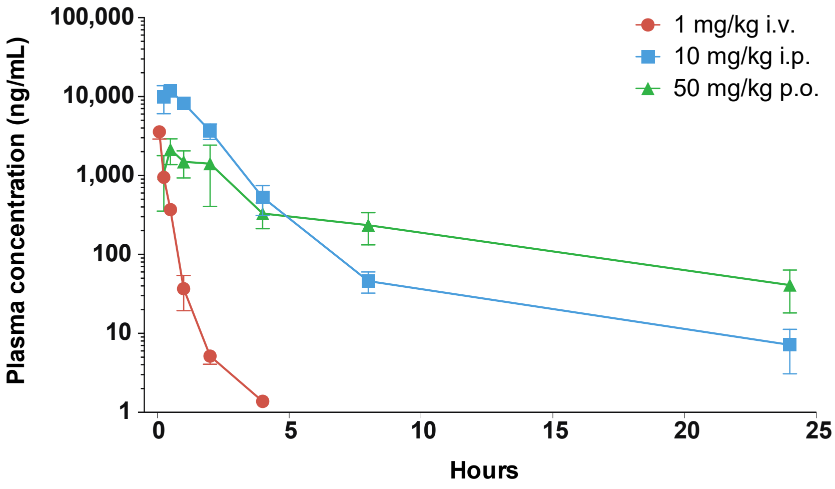

b

TIME POINTS	BRAIN/PLASMA	LIVER/PLASMA
4h	0.50 ± 0.24	3.75 ± 1.06
8h	3.14 ± 0.74	6.32 ± 2.23
12h	BLQ	BLQ

Figure S6. Pharmacokinetic profiling and concentrations in specific tissues of FHP01. **(a)** Pharmacokinetic profiling of FHP01 plasma concentrations after intra-venous (i.v.), intra-peritoneal (i.p.) and per os (p.o.) administration. Mean (±SD) plasma concentration vs. time profile of FHP01 following administration at 1.0 mg/kg, 10 mg/kg and 50 mg/kg to Swiss Albino mice. **(b)** FHP01 tissue concentration after i.p. administration. Drug levels in the brain and in the liver (expressed in ng/g) were normalized against the levels in plasma, (expressed in ng/mL). BLQ indicates that the ratio was not calculated, as the values were Below the Limits of Quantification in all the tissues.

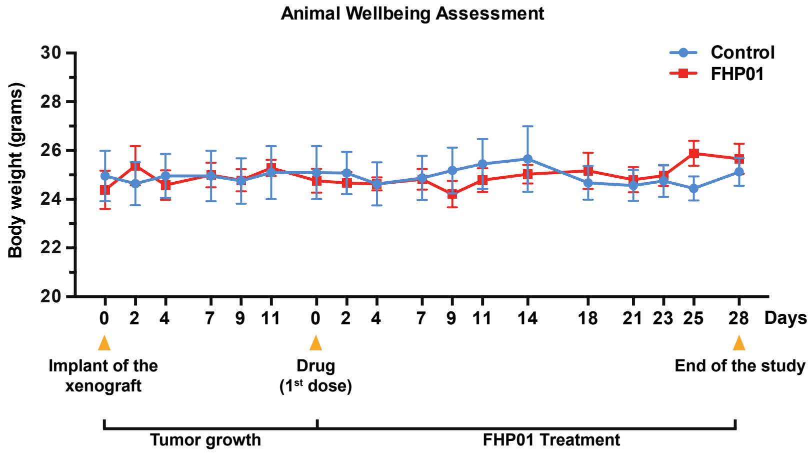

Figure S7. Assessment of animal wellbeing. During the experiment animal weight did not changed significantly. Animal were monitored from their arrival and weighted three times per week. No major signs of discomfort were noted in any of the animals to the end of the procedures. One animal from the control group was sacrificed as the tumor was exceeding the volume limit (accordingly with the humane end point score declared in the animal welfare body guidelines) and taken out from the account. Values are expressed as mean ± SEM in grams (Two Way Anova RM).

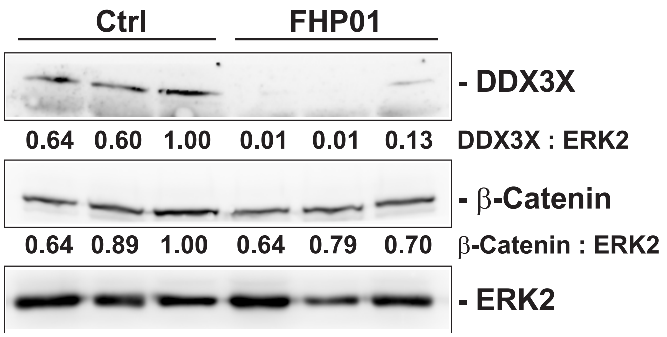

Figure S8. FHP01 reduces DDX3X expression and WNT signaling in tumors from treated mice. Athymic nude mice were injected with MDA MB 231 on both flanks and treated with 45 mg/kg FHP01 suspension i.p. three times per week for 4 weeks. (see, Figure 4a). Then, tumors were collected and representative samples analyzed by WB to score DDX3X and β-catenin protein levels. Anti-ERK2 immunoblot was used to normalize total lysates protein levels. Intensitometric analysis of DDX3X and β-catenin, normalized by ERK2 protein levels, was performed using NIH ImageJ.

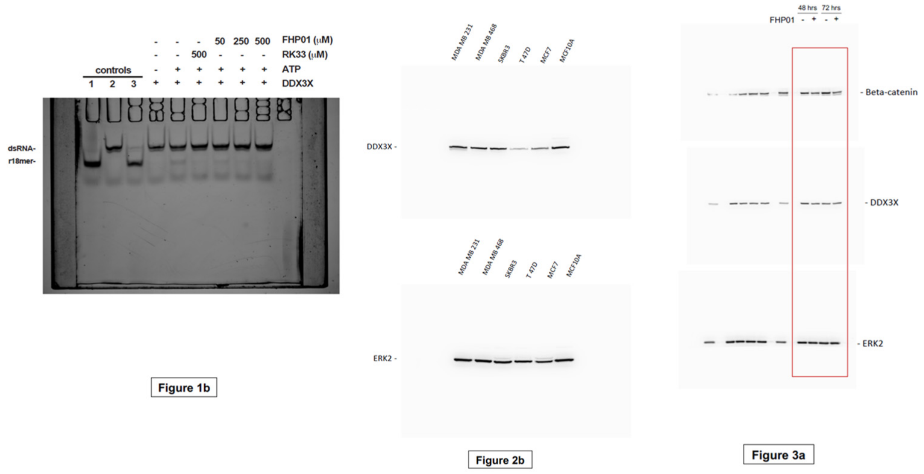

Figure S9. Original Western Blot images.

2. Supplementary Table

Table S1. ARRIVE-study in vivo guideline grid.

1. Study design	In this study n referred to the number of tumors implanted in both flanks of control and treated animals used in the protocol (5 animals for each group) with an equal number of volume acquisition 3 time per week for 4 weeks.
2. Sample size	To calculate the sample size, we based the selection of a large effect size and the standard deviation on previous xenograft experiments in our lab, in vitro preliminary results and finally on relevant literature. The power was set at 80%. G power simulator.
3. Inclusion and exclusion criteria	Animals were purchased by authorized supplier (Charles River Laboratories) and acclimatized for 5 days in standard conditions. Animals were weighed at the start of the procedure. All animals were included in the study showing optimal general condition.
4. Randomization	Randomization method was minimization on the basis of tumor volume at the start of the treatment. Each group contained 5 animals, individually identified.
5. Blinding	No blinding was performed as only one operator was allowed to the protocol of treatment with a patent-protected molecule. Blinding was performed during data analysis, when data from the different groups were blind-coded prior to analysis.
6. Outcome measure	The tumor volume was measured by manual caliper and calculated as 1/2(length × width^2). It represents the principal outcome measure by which statistical effect of DDX3 inhibitor treatment was described. Body weight (g) was also recorded to monitor animal wellbeing and general conditions.
7. Statistic methods	The outcome measure was the tumor volume: the two independent variables were treatment and time. The two-way RM ANOVA method was used to describe the effect of the DDX3 inhibitor treatment versus time. Each animal underwent repeated measure during the whole length of the experiment. Significance was set at $p < 0.05$. For the control group we analyzed 8 experimental units from 4 animals (one animal was sacrificed as for HEP and taken out of the account), 9 units from treated animals (one outlier). Analysis was performed using Prism software.
8. Experimental animals	Six-week-old athymic nude NU/NU CD1 mice were purchased from Charles Rivers Laboratories. Five animals in each cage acclimatized in our animal facilities for 5 days in individually ventilated cages. Animals were fed with the lab diet ad libitum and water was accessible at all times and kept under standard specific-pathogen-free conditions of 12h light/dark cycle. Observational analysis was performed at the beginning of experimental procedure to ensure animal wellbeing.
9. Experimental procedure	To test the anticancer effect of FHP01, a DDX3X inhibitor, a 45 mg/kg solution of the agent was intra peritoneal injected in MDA MB 231 human breast cancer cell NU/NU xenograft mouse model to reduce tumor growth. After acclimatization of 5 days, general conditions of the animal were observed. MDA MB 231 cells (2.8 × 10^6) for the inoculum were suspended in 150 μl PBS 1:1 Matrigel and injected subcutaneously on both size of the flank region of nude mice. Animals were then left to recuperate in their original cages and daily monitored until the mass volume reached the mean value of *circa* 25 mm^3 (25.42 ± 2.02 mm^3). After minimization according to tumor volume, treatment started using 100 μl of a 45 mg/kg FHP01 suspended in a vehicle consisting of 10% DMSO, 5% Tween 80, 85% H_2O via intra-peritoneal injection three times per week, for 4 weeks. In parallel, controls received 100 μl of vehicle injection (10% DMSO, 5% Tween 80, 85% H_2O). Gently handling the animals, manual caliper was used to calculate each tumor volume as ½ (length × width^2). At the end of the procedures, euthanasia was performed using isofluorane to anesthetize and, finally, CO_2. Internal organs were collected for further analysis. All the different phases of

	procedures were performed during light period and each procedure lasted less than 60 minutes.
10. Results	FHP01 treatment exerted a significant *in vivo* anti-tumor activity when compared to the control group. Specifically, at Day 0 of the treatment, mean tumor volume of the FHP01 group (25.49 ± 4.58 mm^3) was similar to that in the vehicle group (24.01 ± 3.64 mm^3). Conversely, starting from Day 18, tumor volumes were significantly different between the two treatment groups (24.18 ± 4.44 mm^3 in the FHP01 group vs. 73.40 ± 9.72 mm^3 in the control group). This difference continued to increase until the end of the experiment at Day 28 (37.23 ± 11.45 mm^3 in the FHP01 group vs. 129.85 ± 21.39 mm^3 in the control group). No loss of animal body weight was detected during all the treatment period indicating a general state of good health of all included animals with an overall excellent biocompatibility of the drug and a good tolerance of nude mice to the dose of 45 mg/kg.<br>The effect size calculated at the end of the procedure was about 4-fold decrease in tumor growth (control/treated) with a confidence interval of 95%.

3. Supplementary Materials and Methods

3.1. Chemistry

The commercially available reagents were purchased by Sigma-Aldrich and used without further purification. Prior to use solvents (DCM and MeOH) were dried by distillation from magnesium methoxyde or calcium hydride. TLC was carried out using Merck TLC plates silica gel 60 F254. Chromatographic purifications were performed using Waters Acquity UPLC with Single Quadrupole Detector with ESI source, and XBridge BEH C18 XP column. 1H-NMR spectra were recorded at 300 MHz, on a Mercury 300 NMR spectrometer. Chemical shifts are reported relative to tetramethylsilane at 0.00 ppm. 1H patterns are described using the following abbreviations: s = singlet, d = doublet, t = triplet, q = quartet, quin = quintet, sx = sextet, sept = septet, m = multiplet, br = broad signal, br s = broad singlet.

Mass spectra (MS) data were obtained using an Agilent 6100 LC/MS (G6130B) spectrometer with a 0.4 mL/min flow rate using a binary solvent system of 95:5 methanol/water. UV detection was monitored at 215 nm and 276 nm. Mass spectra were acquired in positive and negative mode scanning over the mass range.

4-butyl-1-(4-nitrophenyl)-1H-1,2,3-triazole (1b). (Purification eluent: DCM/MeOH 95:5). Yield 94%, yellow solid.MS (ESI) m/z 245 [M-H]-, 281 [M+Cl]-.

4-butyl-1-(4-aminophenyl)-1H-1,2,3-triazole (1c) (Purification eluent: DCM/MeOH 95:5). Yield 91%, white solid. MS (ESI) m/z 217 [M+H]+, 240 [M+Na]+.

1-(4-(4-butyl-1H-1,2,3-triazol-1-yl)phenyl)-3-(2-(trifluoromethyl)phenyl)urea (FHP01): (Purification eluent: H_2O/ACN 70:30).Yield 40%, white solid. HPLC Purity 99.7% (at 215nm); 1HNMR (400 MHz DMSO-d6):δ 9.60 (s, 1H), 8.47 (s, 1H), 8.16 (s, 1H), 7.95 (d, 1H), 7.79 (d, 2H), 7.74-7.62 (m, 4H), 7.30 (t, 1H), 2.69 (t, 2H), 1.65 (quint, 2H), 1.37 (sx, 2H), 0.92 (t, 3H)ppm. MS (ES+) m/z 404.8 [M+1], (ES-) m/z 402.9 [M-1].

3.2. Homology Modeling

For the present computational studies, the closed conformation that binds double stranded RNA is fundamental, and the models of the closed conformation of DDX3X has been prepared by homology modeling using the Swiss-Model web Server [3].

The homology model was generated by SWISS-MODEL web interface. The template library (SMTL version 2017-05-03, PDB release 2017-04-28) was searched with Blast [4] and HHBlits [5] for evolutionary related structures matching the targets sequence. The global and per-residue model quality was assessed using the QMEAN scoring function [6]. Finally, for each helicase, the best QMEAN score homology model of closed conformation was selected for further analysis by molecular dynamics simulations.

The homology model for DDX3X was built with ProMod3 (version 1.0.2, OpenStructure, SIB Swiss Institute of Bioinformatics Biozentrum University of Basel, Basel, Switzerland) [7] the protein was modeled as monomer without ligands with a GMQE value of

0.51 (per residue local quality estimation) (Figure S10a) and a QMEAN value of -1.80 (Figure S10b).

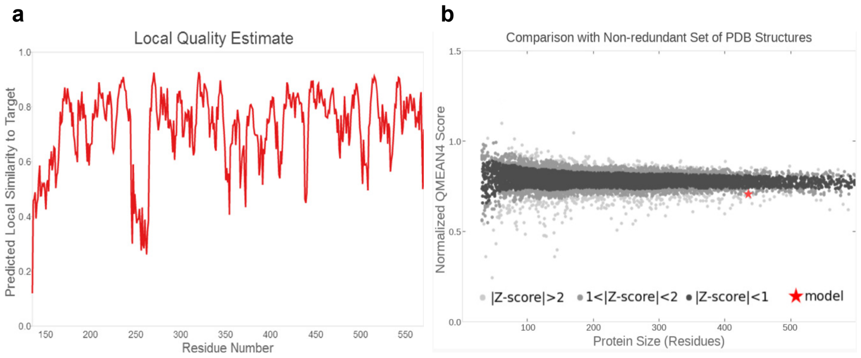

Figure S10. Homology Modeling. **a**) GMQE index per residue for the DDX3X homology model. **b**) Comparison of normalized QMEAN score (star) of the DDX3X homology model with the distribution of normalized QMEAN score for a non-redundant set of PDB structures.

The template structure selected was the ATP dependent RNA helicase vasa, the X-Ray structure with PDB id 2DB3 (chain B), resolution 2.20 Å, residue range 136-671, coverage of target sequence 63%, with a sequence identity of 51.32%, and sequence similarity score 0.44, identified by BLAST (Figure S11).

```
Target     MSHVAVENALGLDQQFAGLDLNSSDNQSGGSTASKGRYIPPHLRNREATKGFYDKDSSGWSSSKDKDAYSSFGSRSDSRG
2db3.1.B   --------------------------------------------------------------------------------
Target     KSSFFSDRGSGSRGRFDDRGRSDYDGIGSRGDRSGFGKFERGGNSRWCDKSDEDDWSKPLPPSERLEQELFSGG-NTGIN
2db3.1.B   -------------------------------------------------------YIPPEPSNDAIE--IFSSGIASGIH
Target     FEKYDDIPVEATGNNCPPHIESFSDVEMGEIIMGNIELTRYTRPTPVQKHAIPIIKEKRDLMACAQTGSGKTAAFLLPIL
2db3.1.B   FSKYNNIPVKVTGSDVPQPIQHFTSADLRDIIIDNVNKSGYKIPTPIQKCSIPVISSGRDLMACAQTGSGKTAAFLLPIL
Target     SQIYSDGPGEALRAMKENGRYGRRKQYPISLVLAPTRELAVQIYEEARKFSYRSRVRPCVVYGGADIGQQIRDLERGCHL
2db3.1.B   SKLLED-PHEL-----ELGR-------PQVVIVSPTRELAIQIFNEARKFAFESYLKIGIVYGGTSFRHQNECITRGCHV
Target     LVATPGRLVDMMERGKIGLDFCKYLVLDEADRMLDMGFEPQIRRIVEQDTMPPKGVRHTMMFSATFPKEIQMLARDFLDE
2db3.1.B   VIATPGRLLDFVDRTFITFEDTRFVVLDEADRMLDMGFSEDMRRIMTHVTMRPE--HQTLMFSATFPEEIQRMAGEFLKN
Target     YIFLAVGRVGSTSENITQKVVWVEESDKRSFLLDLLNATGKDSLTLVFVETKKGADSLEDFLYHEGYACTSIHGDRSQRD
2db3.1.B   YVFVAIGIVGGACSDVKQTIYEVNKYAKRSKLIEILSEQADG--TIVFVETKRGADFLASFLSEKEFPTTSIHGDRLQSQ
Target     REEALHQFRSGKSPILVATAVAARGLDISNVKHVINFDLPSDIEEYVHRIGRTGRVGNLGLATSFFN-ERNINITKDLLD
2db3.1.B   REQALRDFKNGSMKVLIATSVASRGLDIKNIKHVINYDMPSKIDDYVHRIGRTGRVGNNGRATSFFDPEKDRAIAADLVK
Target     LLVEAKQEVPSWLENMAYEHHYKGSSRGRSKSSRFSGGFGARDYRQSSGASSSSFSSSRASSSRSGGGGHGSSRGFGGGG
2db3.1.B   ILEGSGQTVPDFL-------------------------------------------------------------------
Target     YGGFYNSDGYGGNYNSQGVDWWGN
2db3.1.B   ------------------------
```

Figure S11. Sequence alignment of the DDX3X sequence (Target) with the template sequence (2db3.1.B).

3.3. Molecular Dynamics Simulations

The selected homology model of DDX3X has been prepared for a series of explicit solvent molecular dynamics simulations. The homology model was refined by adding ATP co-factor and magnesium 2+ ion with coordination water molecules, the protonation state and orientation of histidine, asparagine and glutamine residues have been optimized by PDB2PQR (version 2.1.0, Poissonboltzmann; Washington University in St. Louis, St. Louis, MO, USA); [8, 9]. All the missing hydrogen atoms were added according to the AMBER 14 topology parameters using the LEAP module of AMBERTOOLS. ATP was parametrized using the parameters set as described from Meagher, Redman and Carlson [10]. The proteins were solvated in a cubic water-box of TIP3P waters, solvent constituting 10 Å buffer form the protein to the periodic boundary. The system was then neutralized adding the appropriate number of sodium and chloride ions to simulate a concentration of 0.1M. Topology files were parameterized using AMBER14SB forcefield.

All the molecular dynamics simulations were performed with NAMD (version 2.12, Theoretical and Computational Biophysics Group; University Of Illinois at Urbana-Champaign, Urbana, IL, USA) [11]. A total of 5000 steps of energy minimization have been carried out to remove artificial contacts, without constraint. The system was then equilibrated for 1 ns with a 2 fs time-step at 1 atm pressure following a two-step equilibration protocol:

the first step consist of 0.1 ns simulation where the system was heated form 0°K to 310°K with a Berendsen thermostat simulation; the second step was a 0.9 ns Langevin dynamics simulation at 310°K temperature. Finally, a 20 ns simulation was carried out. The simulations were performed with GPU accelerated molecular dynamics in an 88 CPUs system with two GPU (NVIDIA GeForce GTX 1080) with an average speed of 0.2 days/ns of simulation. Simulation snapshots have been collected every 10,000 steps resulting in 1000 frames, that represent the conformational sampling of the aminoacids sidechains degree of freedom, in Figure S12 are reported the RMSD fluctuation plots of the carbon alpha trace of the protein respect to the average structure, indicating that the helicases modeled was reasonably equilibrated.

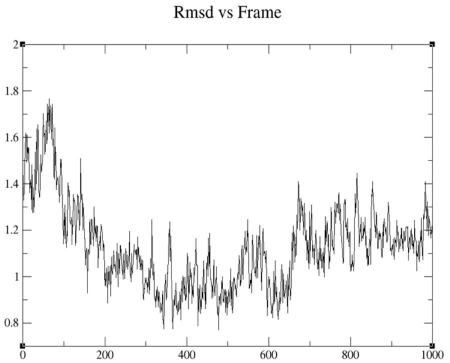

Figure S12. RMSD variation of carbon alpha trace during the Molecular dynamics simulation of DDX3X.

The molecular dynamics trajectories have been sampled to select a series of 20 frame representative of the flexibility of binding site residues, by clustering and centroid conformation selection.

The pairwise RMSD matrix between the frames has been calculated with VMD [12], by alignment and RMSD calculation between the residues that contribute to the helicase binding site surface (Figure S13).

Protein ID	Residue ID and sequence																									
DDX3X (O00571 version 3)	273	274	275	276	277	278	301	302	303	304	306	323	324	325	326	327	328	329	330	348	350	351	352	354	355	356
	A	P	T	R	E	L	Y	G	G	A	I	T	P	G	R	L	V	D	M	E	D	R	M	D	M	G

Protein ID	Residue ID and sequence																									
DDX3X (O00571 version 3)	357	358	360	384	448	449	450	451	472	473	480	498	499	500	501	502	503	504	505	518	519	520	521	523	524	527	528
	F	E	Q	T	V	E	T	K	H	G	R	T	A	V	A	A	R	G	L	L	P	S	D	E	E	H	R

Figure S13. List of residues composing the RNA binding site DDX3X. The sequence of the protein has been retrieved by UniProtKB database (https://www.uniprot.org/uniprot/ accessed on 19 October 2018) in fasta format with the following accession code, DDX3X (O00571, version 3).

The clustering was performed by hierarchical clustering with average linkage at RMSD 2.0 Å selecting a maximum number of 20 clusters (Figure S14).

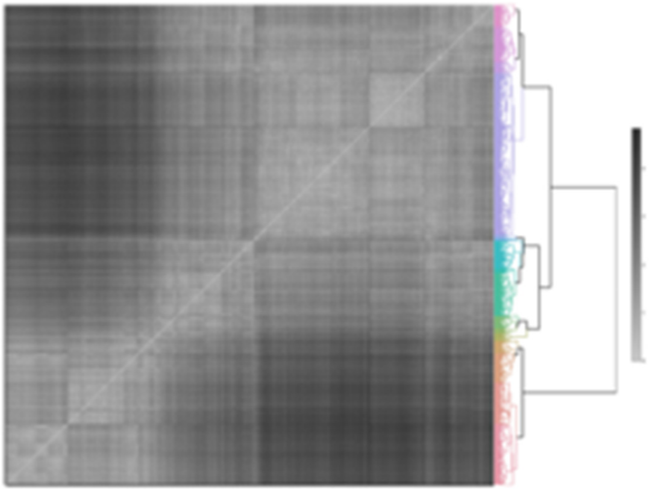

Figure S14. Sorted RMSD distance matrix between conformations of DDX3X obtained from the molecular dynamic simulation.

For each protein the representative structure of each cluster was selected for the ensemble molecular docking procedure [13] to account the flexibility of the binding site.

3.4. Ensemble Molecular Docking

The molecular docking experiments were performed combining two different programs SMINA [14] and RBDOCK [15, 16]. The molecular docking protocol is based on the docking in parallel with both SMINA and RBDOCK, in the conformation ensemble of DDX3X. The ligand docking poses obtained from the two docking programs were rescored using an external scoring function, RF-SCORE-VS [2], and clustered by hierarchical clustering with average linkage at RMSD 2.5 Å.

Key interaction of FHP01 have been identified using the following parameters: the hydrogen bond donor (HBD) and hydrogen bond acceptor (HBA) interaction are identified when the distance between the heavy atoms is less or equal than 3.6 Å and a maximum angle of 63 degree, the Van der Waals interactions (VdW) are considered present when any of the atoms of the ligand are at a distance between 3 and 4.5 Å from the atom of the protein residues.

3.5. Synthesis of FHP01

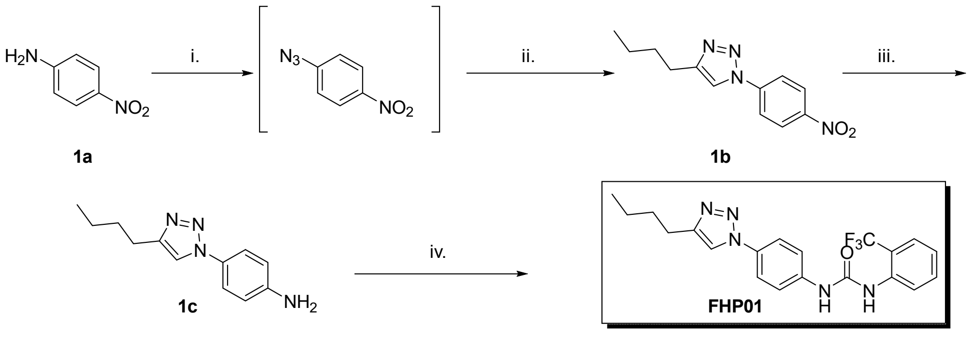

Reagents and conditions: i. a) tBuONO, CH_3CN, 0°C, 20 min, b) TMSN3, CH_3CN, r.t., 2h; ii. 1-Hexyne $CuSO_4$. $5H_2O$, Sodium Ascorbate, H_2O:tBuOH (1:1 v/v), MW 125°C, 15 min; (94%); iii. Raney nickel, hydrazine, MeOH, r.t. (91%); iv. 1-isocyanato-2-(trifluoromethyl)benzene, DCM, 5h, r.t. (40%).

The synthesis procedure followed to obtain FHP01 is shown in Scheme 1. The intermediate 1-azido-4-nitrobenzene was obtained from 4-nitroaniline (1a), by reaction with tBuONO in acetonitrile in ice bath for 20 min, then ice bath was removed and TMSN3 was

added and reacted at room temperature for 2 hours. The 1-azido-4-nitrobenzene was reacted with 1-Hexyne in presence of $CuSO_4$ pentahydrate and sodium Ascorbate in H_2O/tBuOH (1:1 v/v) in microwave at 125 °C for 15 min, leading to obtain the 4-butyl-1-(4-nitrophenyl)-1H-1,2,3-triazole (1b). The intermediate 1c was obtained by reduction with Raney nickel and hydrazine hydrate, refluxing in methanol for 3 hours. The final compound FHP01 was afforded by reaction with 1-isocyanato-2-(trifluoromethyl)benzene in DCM for 5 hours at room temperature. FHP01 was purified by reverse phase chromatography (Water/Acetonitrile), obtaining 400 mg as white solid with purity of 99.7% (at 215 nm) and 99.6% (at 276 nm).

4. Supplementary References

1. Brai, A., Fazi, R., Tintori, C., Zamperini, C., Bugli, F., Sanguinetti, M., Stigliano, E., Este, J., Badia, R., Franco, S. et al. Human DDX3 protein is a valuable target to develop broad spectrum antiviral agents. *Proc Natl Acad Sci U S A* **2016**, 113, 5388–5393.
2. Wójcikowski, M., Ballester, P. J., and Siedlecki, P. Performance of machine-learning scoring functions in structure-based virtual screening. *Sci Rep* **2017**, 7, 46710.
3. Waterhouse, A., Bertoni, M., Bienert, S., Studer, G., Tauriello, G., Gumienny, R., Heer, F. T., de Beer, T. A. P., Rempfer, C., Bordoli, L. et al. SWISS-MODEL: homology modelling of protein structures and complexes. *Nucleic Acids Res* **2018**, 46, W296–W303.
4. Altschul, S. F., Madden, T. L., Schäffer, A. A., Zhang, J., Zhang, Z., Miller, W., and Lipman, D. J. Gapped BLAST and PSI-BLAST: a new generation of protein database search programs. *Nucleic Acids Res* **1997**, 25, 3389–3402.
5. Remmert, M., Biegert, A., Hauser, A., and Söding, J. HHblits: lightning-fast iterative protein sequence searching by HMM-HMM alignment. *Nat Methods* **2011**, 9, 173–175.
6. Benkert, P., Biasini, M., and Schwede, T. Toward the estimation of the absolute quality of individual protein structure models. *Bioinformatics* **2011**, 27, 343–350.
7. Biasini, M., Schmidt, T., Bienert, S., Mariani, V., Studer, G., Haas, J., Johner, N., Schenk, A. D., Philippsen, A., and Schwede, T. OpenStructure: an integrated software framework for computational structural biology. *Acta Crystallogr D Biol Crystallogr* **2013**, 69, 701–709.
8. Dolinsky, T. J., Nielsen, J. E., McCammon, J. A., and Baker, N. A. PDB2PQR: an automated pipeline for the setup of Poisson-Boltzmann electrostatics calculations. *Nucleic Acids Res* **2004**, 32, W665–7.
9. Dolinsky, T. J., Czodrowski, P., Li, H., Nielsen, J. E., Jensen, J. H., Klebe, G., and Baker, N. A. PDB2PQR: expanding and upgrading automated preparation of biomolecular structures for molecular simulations. *Nucleic Acids Res* **2007**, 35, W522–5.
10. Meagher, K. L., Redman, L. T., and Carlson, H. A. Development of polyphosphate parameters for use with the AMBER force field. *J Comput Chem* **2003**, 24, 1016–1025.
11. Phillips, J. C., Braun, R., Wang, W., Gumbart, J., Tajkhorshid, E., Villa, E., Chipot, C., Skeel, R. D., Kalé, L., and Schulten, K. Scalable molecular dynamics with NAMD. *J Comput Chem* **2005**, 26, 1781–1802.
12. Humphrey, W., Dalke, A., and Schulten, K. VMD: visual molecular dynamics. *J Mol Graph* **1996**, 14, 33–8, 27.
13. Amaro, R. E., Baudry, J., Chodera, J., Demir, Ö., McCammon, J. A., Miao, Y., and Smith, J. C. Ensemble Docking in Drug Discovery. *Biophys J* **2018**, 114, 2271–2278.
14. Trott, O., and Olson, A. J. AutoDock Vina: improving the speed and accuracy of docking with a new scoring function, efficient optimization, and multithreading. *J Comput Chem* **2010**, 31, 455–461.
15. Morley, S. D., and Afshar, M. Validation of an empirical RNA-ligand scoring function for fast flexible docking using Ribodock. *J Comput Aided Mol Des* **2004**, 18, 189-208.
16. Ruiz-Carmona, S., Alvarez-Garcia, D., Foloppe, N., Garmendia-Doval, A. B., Juhos, S., Schmidtke, P., Barril, X., Hubbard, R. E., and Morley, S. D. rDock: a fast, versatile and open source program for docking ligands to proteins and nucleic acids. *PLoS Comput Biol* **2014**, 10, e1003571.
